# Determinants and Prognostic Value of Persistent Congestion Assessed by Venous Excess Ultrasound Score in Acute Heart Failure

**DOI:** 10.3390/diagnostics16203374

**Published:** 2026-10-16

**Authors:** Emmanouela Peteinidou, Marino Basha, Stylianos Daios, Vasileios Anastasiou, Evdoxia Stavropoulou, Christoforos Tsamtzidis, Eleftheria Baltagianni, Emmanouil Fardoulis, Christos Mantzios, Matthaios Didagelos, Antonios Ziakas, Vasileios Kamperidis

**Affiliations:** 1st Department of Cardiology, School of Medicine, Faculty of Health Sciences, AHEPA University Hospital, Aristotle University of Thessaloniki, St. Kiriakidi 1, GR 54636 Thessaloniki, Greeceevdostavrop@gmail.com (E.S.); christopher.tsam@gmail.com (C.T.); b.elira@hotmail.com (E.B.);

**Keywords:** VExUS score, congestion, acute heart failure, tricuspid regurgitation, outcome, rehospitalization, survival

## Abstract

**Background:** Reliable quantification of systemic venous congestion in acute heart failure (HF) has become feasible with the advent of the venous excess ultrasound (VExUS) score, informing therapeutic management. Nevertheless, the determinants of the VExUS score trajectory during hospitalization and the prognostic implications of persistent subclinical congestion at discharge remain poorly defined. **Methods:** Consecutive patients admitted for acute HF were prospectively enrolled (*n* = 418, 73.3 ± 11.8 years old, 59.1% male). VExUS scores were assessed at admission and prior to discharge to categorize the patients into three groups: non-congested (persistent score of 0; *n* = 81), improved (score reduction of ≥1 point; *n* = 214), and non-improved (persistent congestion without score reduction; *n* = 123). Baseline clinical data were collected and all the patients had a transthoracic echocardiogram on admission. All patients were followed up post-discharge and the primary endpoint was a composite of all-cause mortality or HF hospitalization. **Results:** Patients with non-improved VExUS exhibited a higher burden of right-sided remodeling and dysfunction including larger right chambers, lower right ventricle (RV) systolic function indices, higher RV systolic pressures and a higher prevalence of severe tricuspid regurgitation (all *p* < 0.001). Severe tricuspid regurgitation was the sole independent predictor of VExUS non-improvement (OR 2.86, 95% CI 1.51–5.45, *p* = 0.001). Non-improved VExUS (HR 1.77; 95% CI 1.17–2.67; *p* = 0.007), severe tricuspid regurgitation (HR 1.88; 95% CI 1.16–3.02; *p* = 0.010) and intravenous furosemide dose (HR 1.00; 95% CI 1.00–1.01; *p* = 0.011) were independent predictors of the composite primary endpoint. Inclusion of non-improved VExUS provided incremental prognostic value over models including clinical, laboratory, and echocardiographic parameters (Δχ^2^= 9.22; *p* = 0.007). **Conclusions:** VExUS non-improvement throughout hospitalization marks patients with worse primary endpoint Severe tricuspid regurgitation emerges as an independent determinant of VExUS non-improvement which is independently associated with poor long-term outcomes.

## 1. Introduction

Congestion is a defining diagnostic feature of heart failure (HF) with a well-established correlation to adverse clinical outcomes [1]. Its assessment was imprecise, owing to the invasive character of direct right atrial pressure measurement, while noninvasive assessment through inferior vena cava (IVC) diameter has proven unreliable [2]. Recently, the venous excess ultrasound (VExUS) score has emerged as a readily accessible modality that provides a comprehensive insight into the congestion status of patients, demonstrating a robust correlation with invasively measured right atrial pressures [3,4].

Congestion has an integral role in patient management since it is the main therapeutic target during hospitalization for acute HF to relieve the patients’ symptoms that were the cause of the admission. However, the absence of a standardized framework to guide decongestive strategies puts the patient at risk of being discharged clinically euvolemic but still congested according to the VExUS score, signifying a worse prognosis [5]. There is some scarce data about the use of the VExUS score to guide decongestion, with unknown clinical implications [6].

The dynamic changes in VExUS score from admission to discharge have not been thoroughly studied, including the forces behind the imaging-based congestion despite clinical euvolaemia. Herein, this study sought to examine the VExUS trajectory from admission to discharge, to identify factors associated with VExUS non-improvement despite decongestion pharmacotherapy and to evaluate the prognostic utility of VExUS dynamic changes.

## 2. Materials and Methods

### 2.1. Study Population

All consecutive patients admitted to the cardiology department of AHEPA University General Hospital in Thessaloniki, Greece, from April 2022 until August 2024 for acute HF were enrolled as part of the prospective Beyond-Myo HF Study (ClinicalTrials.gov identifier: NCT05573997; https://www.clinicaltrials.gov/study/NCT05573997, accessed on 23 August 2026) [7]. Inclusion and exclusion criteria are summarized in the Beyond-Myo HF Study Protocol. In brief, all adult patients with (1) symptoms and/or signs of HF, (2) abnormal N-terminal pro B-type natriuretic peptide (NT-proBNP) levels and (3) echocardiographic evidence of cardiac structural and/or functional abnormalities evaluated within 24 h of admission were included. Patients on mechanical ventilation were excluded due to their inability to follow breathing instructions and in order to avoid the interaction with the mechanical ventilation parameters [8]. Anthropometric, biochemical, and clinical characteristics along with pharmacological treatment in the acute phase of hospitalization were documented. The study’s endpoint was the composite of HF rehospitalization and all-cause mortality. This investigation conforms with the principles outlined in the Declaration of Helsinki (2013 amendment) and the study protocol was approved by the Ethics Committee of the School of Medicine of Aristotle University of Thessaloniki (approval no. 19/2022).

### 2.2. Venous Congestion Assessment

Systemic venous congestion was evaluated in all patients using the VExUS grading system. Serial examinations were conducted using ultrasound systems equipped with phased-array transducers (Vivid E95 or Vivid S70, GE Healthcare, Chicago, IL, USA), within 24 h of admission and 24 h prior to discharge. The VExUS interrogation was performed in accordance with the original description by Beaubien-Souligny et al. [9] through the integrated assessment of four venous parameters: IVC diameter and pulsed-wave Doppler flow patterns of the hepatic, portal, and intrarenal veins. All examinations were conducted by two investigators, blinded to the clinical and outcome data of all participants, and trained through a dedicated 3-month program in the hospital’s radiology department, with emphasis on renal and portal venous Doppler assessment.

IVC, hepatic vein, and portal vein Doppler assessments were conducted with patients in the dorsal decubitus position. The IVC diameter was measured at end-expiration, perpendicular to the vessel’s long axis, just proximal to the hepatic vein confluence and 0.5–3.0 cm from the right atrial ostium [10]. Hepatic vein Doppler flow was obtained from the subcostal view, with the pulsed-wave Doppler beam aligned parallel to a hepatic vein draining into the IVC. Portal vein flow was assessed from a posterior axillary approach, with vessel identification confirmed by portal venous flow signature (continuous or biphasic) using pulsed-wave Doppler. Intrarenal venous flow was evaluated with the patient in the left lateral decubitus position. The transducer was positioned along the midaxillary line at the 10th intercostal space to obtain a longitudinal view of the right kidney. Color Doppler settings were adjusted to low-velocity scales (<20 cm/s) to facilitate identification of interlobar veins, which typically appeared with flow directed away from the transducer. The vein with an optimal insonation angle was sampled using pulsed-wave Doppler and venous origin was confirmed by waveform characteristics [11].

All Doppler measurements were acquired at end-expiration with simultaneous electrocardiographic recording to allow accurate cardiac cycle phase tracking. An IVC diameter ≥ 20 mm was considered dilated. Hepatic, portal, and renal venous flows were classified as normal, mildly abnormal, or severely abnormal based on their Doppler waveform patterns. Normal hepatic vein flow was defined by systolic wave predominance, while diastolic predominance was considered mildly abnormal, and systolic flow reversal was considered severely abnormal. Portal vein pulsatility was quantified using the pulsatility fraction: (peak velocity-nadir velocity/peak velocity × 100). Values < 30% were considered normal, 30–49% mildly abnormal, and ≥50% severely abnormal. Interlobar renal venous flow was classified as continuous (normal), biphasic (mildly abnormal), or monophasic with diastolic-only flow (severely abnormal), reflecting the downstream effects of progressively elevated central venous pressure on renal venous congestion [12].

Grading was performed under a generally accepted framework [9]: a normal IVC diameter (<20 mm) corresponded to a VExUS score of 0, indicating no venous congestion. When IVC dilation was present (≥20 mm), hepatic, portal, and renal venous Doppler waveforms were assessed: normal or mildly abnormal patterns yielded a score of 1; one severely abnormal waveform yielded a score of 2; and two or more severely abnormal waveforms yielded a score of 3. VExUS scores of 0, 1, 2, and 3 therefore reflected no, mild, moderate, and severe systemic venous congestion, respectively.

Based on the evolution of the VExUS score from admission to discharge, the patients were categorized into three groups: 1. The non-congested, who maintained a VExUS score of 0 throughout hospitalization; 2. The improved, who experienced a reduction of at least one grade in their VExUS score; 3. The non-improved, who had persistent venous congestion without any reduction in VExUS score.

### 2.3. Echocardiographic Analysis

All patients underwent a comprehensive transthoracic echocardiography within 24 h of admission. All echocardiographic studies were prospectively performed by certified cardiologists with high-end ultrasound systems (Vivid E95 or Vivid S70, GE Healthcare). Electrocardiogram-triggered echocardiographic data were digitally stored in a cine-loop format for offline analysis on a dedicated workstation (EchoPAC version 112.0.1, GE HealthCare, Chicago, IL, USA) by an expert cardiologist blinded to the clinical and outcome data of all participants. The echocardiographic protocol is available in detail in the Supplemental Material.

### 2.4. MAGGIC Risk Score

Baseline clinical risk was assessed using the Meta-Analysis Global Group in Chronic Heart Failure (MAGGIC) risk score, a validated prognostic model developed from pooled individual-patient data encompassing diverse HF populations [13]. The MAGGIC score integrates key demographic, clinical, laboratory, and treatment-related variables to estimate mortality risk across the spectrum of HF severity; specifically it includes: age, left ventricular ejection fraction, systolic blood pressure, body mass index, serum creatinine, NYHA class, gender, smoking status, diabetes mellitus, chronic obstructive pulmonary disease, time since HF diagnosis ≥18 months, and use of β-blockers and Angiotensin-Converting Enzyme Inhibitor (ACEi)/Angiotensin Receptor Blocker (ARB) therapy. Its broad validation across both reduced and preserved ejection fraction phenotypes, along with consistent performance in contemporary cohorts, supports its role as a comprehensive measure of baseline clinical risk [13]. By capturing established determinants of prognosis without reliance on advanced imaging or invasive measurements, the MAGGIC score provides a standardized framework for contextualizing disease severity and outcome risk in patients hospitalized with HF [13].

### 2.5. Statistical Analysis

Categorical variables were reported as frequencies (percentages) and compared with the chi-square test. Continuous variables were expressed as mean ± SD if normally distributed and as median (interquartile range) if they followed a non-normal distribution. Comparisons between the 3 groups were performed with the one-way analysis of variance test using Bonferroni’s post hoc test for normally distributed continuous variables or with the Kruskal–Wallis test for continuous variables not following a normal distribution.

The association of clinical and echocardiographic variables with the non-improved VExUS group was tested using univariable logistic regression analysis, and those that demonstrated statistical significance were entered into a multivariable model. The odds ratio (OR), 95% CI, and *p* value were calculated and reported.

The association of clinical and echocardiographic variables with the composite outcome was evaluated using uni- and multivariable Cox proportional hazards regression analysis. The individual components of the composite endpoint were additionally examined by Kaplan–Meier analysis with log-rank testing. To assess the incremental prognostic value of VExUS non-improvement, nested model comparisons were performed using likelihood ratio testing. The MAGGIC risk score served as the baseline model, enabling adjustment for key clinical confounders while minimizing model overfitting. Additional variables were introduced in a stepwise fashion to evaluate their contribution to model performance. Model 1 consisted of the MAGGIC score with the addition of the NT-proBNP, intravenous inotrope dose, and intravenous furosemide dose. Model 2 expanded upon Model 1 by incorporating echocardiographic parameters, including right ventricular end-diastolic area, right ventricular free wall longitudinal strain, right atrial volume, right atrial reservoir strain, right ventricular systolic pressure, severe left-sided valvular heart disease, and severe tricuspid regurgitation. Model 3, the final model, further included VExUS non-improvement. Incremental prognostic value across models was assessed by changes in the chi-square statistic. The proportional hazards assumption was assessed using scaled Schoenfeld residuals. Model discrimination was quantified by Harrell’s C-index with bootstrap 95% confidence intervals before and after inclusion of VExUS non-improvement.

All tests were two-sided, and *p* < 0.05 was considered statistically significant. Statistical analysis was conducted using SPSS software, version 29 (IBM SPSS Statistics, IBM, Armonk, NY, USA).

## 3. Results

### 3.1. Patients’ Stratification According to VExUS Trajectory

**Figure 1:**
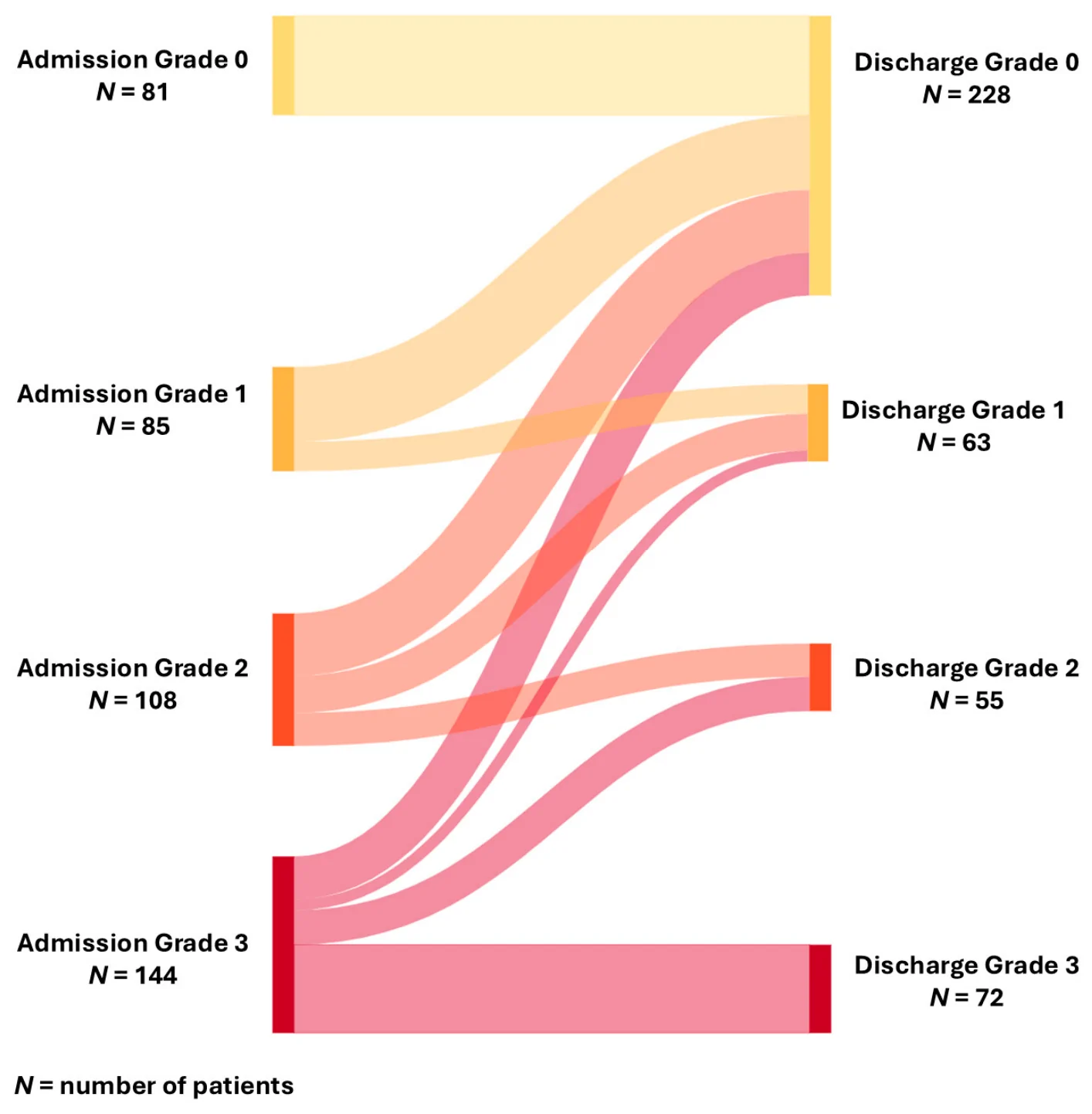
VExUS grade at admission and reassessment. Alluvial plot showing the change in VExUS grade (0–3) from admission (left) to reassessment (right) for the 418-patient cohort.

A total of 418 patients (mean age, 73.3 ± 11.8 years; 59.1% male) were included in the present analysis. Patients were categorized into three groups based on VExUS score trajectory from admission to discharge: the improved group (*n* = 214; 51.19%), reflecting a measurable resolution of venous congestion; the non-improved group (*n* = 123; 29.42%), representing failure to achieve decongestion despite therapy; and the non-congested group (*n* = 81; 19.38%), indicating persistently absent systemic venous congestion. Overall, 214 patients (51.2%) improved by at least one VExUS grade, 123 (29.4%) did not improve, and 81 (19.4%) remained free of congestion throughout. Among those who improved, the reduction was one grade in 119 patients, two grades in 60 and three grades in 35. 147 patients improved to complete resolution of congestion and 67 improved without complete resolution. All 123 non-improved patients had stable grades (1 → 1 in 24, 2 → 2 in 27, 3 → 3 in 72), with no patient deteriorating. The full distribution of admission-to-reassessment transitions is shown in Figure 1.

### 3.2. Clinical and Echocardiographic Characteristics

Patients presented a stepwise escalation of symptoms, MAGGIC score, long-standing HF and chronic kidney disease at admission, from non-congested to improved and non-improved groups (*p* < 0.001). The NT-proBNP biomarker reflected this progressive clinical deterioration, since the levels were higher in both improved and non-improved groups compared with non-congested patients. Table 1 summarizes the baseline demographic and clinical characteristics.

Echocardiographic assessment revealed marked and stepwise worsening structural and functional abnormalities across VExUS trajectories, with the most pronounced derangements observed in patients with non-improved VExUS (Table 2). Right ventricle (RV) and right atrium (RA) adverse remodeling and dysfunction, accompanied by higher RV systolic pressures and a substantially greater prevalence of severe tricuspid regurgitation, were more pronounced in the non-improved group. Severe left-sided valvular heart disease frequency, alongside left atrial dilation and dysfunction and signs of progressively elevated left-sided filling pressures, reinforced the hemodynamic continuum captured by VExUS trajectory groups.

### 3.3. Predictors of VExUS Non-Improvement

Several clinical, biochemical, and echocardiographic parameters were tested with univariable and multivariable logistic regression analysis and only severe tricuspid regurgitation remained an independent predictor of VExUS non-improvement (Table 3).

### 3.4. Predictors of the Composite Outcome

**Figure 2:**
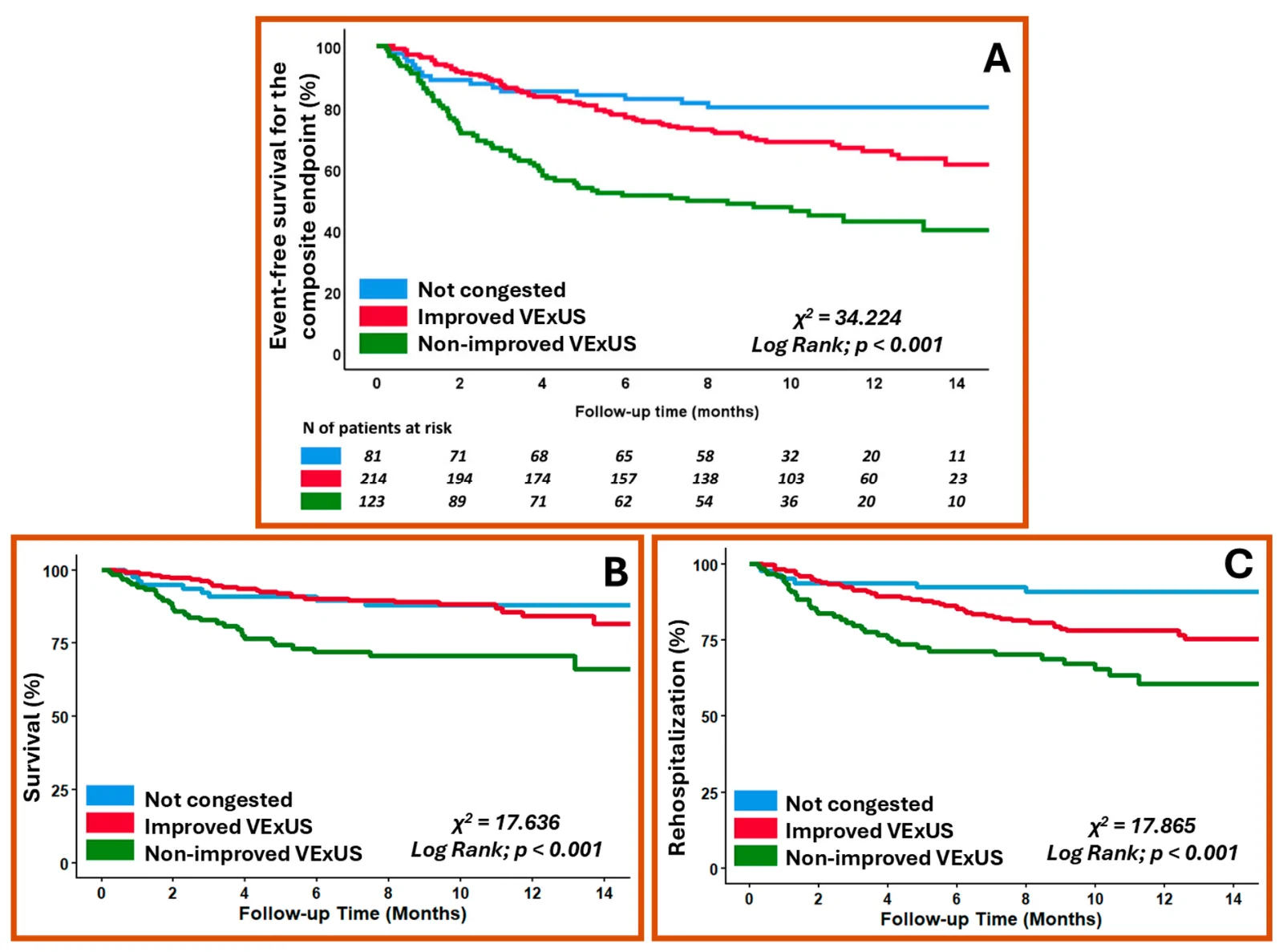
(**A**). Kaplan–Meier event-free survival curves for the composite endpoint among non-congested patients, patients with improved VExUS scores, and patients with non-improved VExUS scores. (**B**). All-cause mortality in the non-congested, improved-VExUS and non-improved-VExUS groups over follow-up. (**C**). Rehospitalization for heart-failure among non-congested, improved-VExUS and non-improved-VExUS groups over follow-up.

A total of 154 (36.84%) patients experienced the composite outcome of all-cause mortality and HF rehospitalization during a follow-up of 8.92 (4.08–11.56) months. The patients in the non-improvement VExUS score group had significantly worse event-free survival among the three groups as demonstrated by the Kaplan–Meier curves (Log-rank *p* < 0.001) (Figure 2). The univariable and subsequent multivariable analysis demonstrated that a non-improved VExUS score was independently associated with the primary composite endpoint (HR 1.77, 95% CI 1.17–2.67, *p* = 0.007) (Table 4).

### 3.5. Incremental Prognostic Value of VExUS Non-Improvement

**Figure 3:**
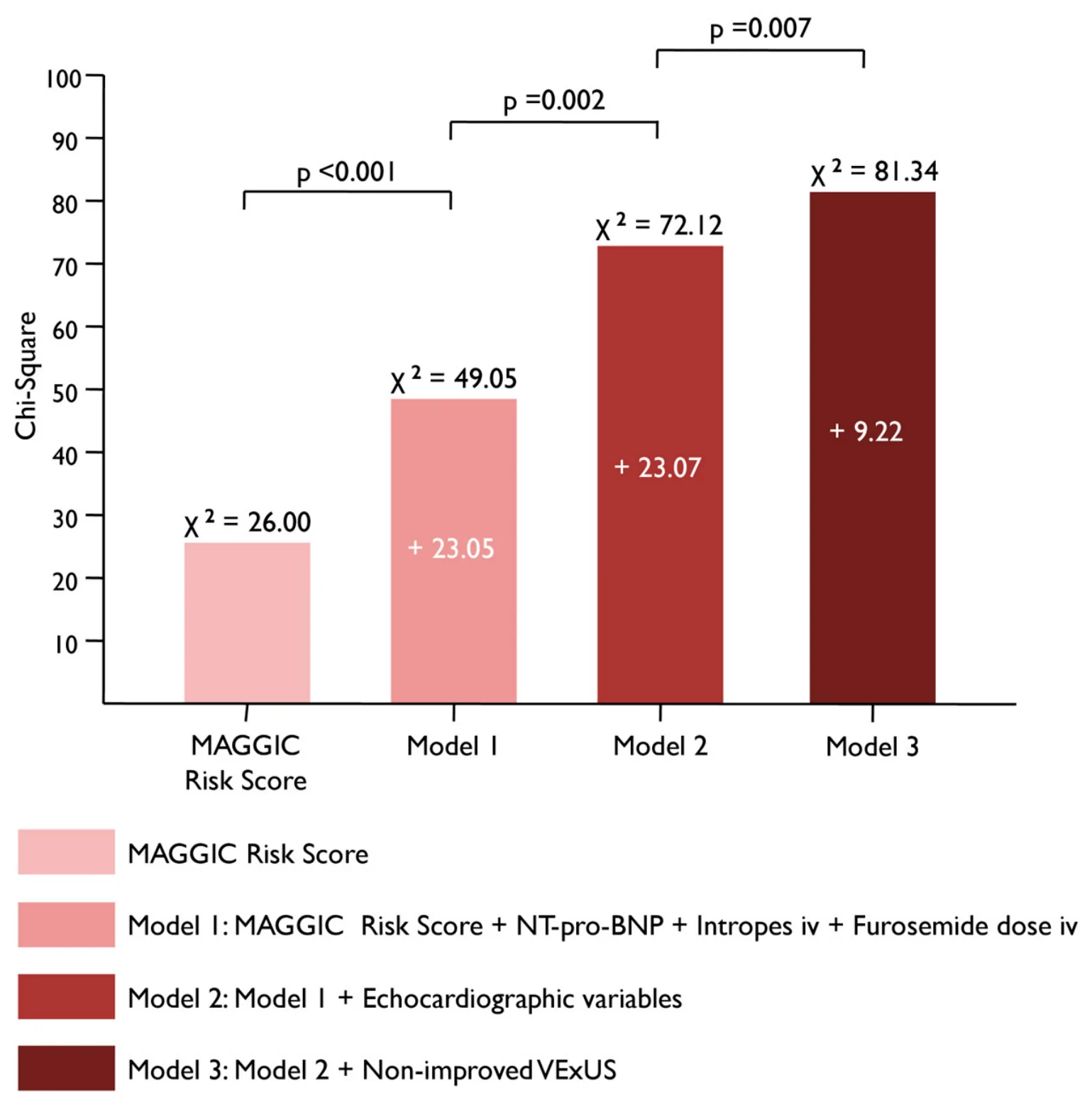
Incremental prognostic value of non-improved VExUS score over MAGGIC Risk Score, clinical and echocardiographic variables. The MAGGIC Risk Score incorporates the following variables: Age, Left ventricular ejection fraction, Systolic blood pressure, Body mass index, Creatinine, NYHA Class, Gender, Smoking, Diabetes, Chronic obstructive pulmonary disease, Heart failure first diagnosed ≥ 18 months, Beta-blocker, and ACEi/ARB. Echocardiographic variables include: Right ventricular end-diastolic area, right ventricular free wall longitudinal strain, right atrial volume, right atrial reservoir strain, right ventricular systolic pressure, Severe left-sided valvular heart disease, Severe tricuspid regurgitation. Abbreviations: ACEi, angiotensin-converting enzyme inhibitor; ARB, angiotensin receptor blocker; NT-proBNP, N-terminal pro B-type natriuretic peptide; NYHA, New York Heart Association; VExUS, venous excess ultrasound score.

To assess whether VExUS non-improvement provided incremental prognostic value beyond established clinical, biochemical and imaging markers, hierarchical Cox regression models were constructed (Figure 3). The baseline model consisting of the MAGGIC risk score yielded a χ^2^ of 26.00. Addition of NT-proBNP, use of intravenous inotropic agents, and intravenous furosemide dose significantly improved model performance (Model 1: χ^2^ = 49.05, *p* < 0.001, Δχ^2^ = +23.05). Further inclusion of a panel of echocardiographic variables (right ventricular end-diastolic area, right ventricular free-wall longitudinal strain, right atrial volume, right atrial reservoir strain, right ventricular systolic pressure, presence of severe left-sided valvular heart disease, and severe tricuspid regurgitation) resulted in a χ^2^ of 72.12 (Model 2, *p* = 0.002, Δχ^2^ = +23.07.) Notably, the addition of VExUS score non-improvement produced a further significant increase in model fit (Model 3: χ^2^ = 81.34, *p* = 0.007, Δχ^2^ = +9.22), indicating that persistent venous congestion at discharge provides independent and incremental prognostic value above clinical risk scores, biochemical markers, in-hospital management, and comprehensive echocardiographic assessment. Model discrimination, assessed by Harrell’s C-index, increased in parallel across the nested models, from 0.632 (95% CI 0.581–0.681) for the MAGGIC-based model to 0.705 (0.656–0.753) after the addition of echocardiographic variables and to 0.721 (0.674–0.768) with the inclusion of VExUS non-improvement (ΔC = 0.017, 95% CI 0.002–0.031, *p* = 0.028).

## 4. Discussion

The longitudinal trajectory of the VExUS score during hospitalization, rather than a single cross-sectional measurement, serves as a potent indicator of clinical phenotype and provides significant prognostic information. Patients who failed to demonstrate improvement of VExUS score represent a vulnerable subgroup, characterized by a markedly compromised clinical and echocardiographic profile. Notably, nearly one-third of patients admitted with congestion (29.4%) did not achieve meaningful resolution of systemic venous congestion by the time of discharge despite receiving guideline-directed decongestive therapy, highlighting the prevalence of untreated subclinical congestion. Severe tricuspid regurgitation is identified as the sole independent predictor of VExUS non-improvement. This association should be interpreted with caution, since severe TR directly generates the hepatic venous systolic reversal that contributes to the score, and is therefore partly inherent to its construction. It also raises the possibility that persistent congestion in these patients reflects, beyond diuretic titration alone, the right heart–pulmonary vasculature mechanics associated with the regurgitation. Furthermore, a non-improved VExUS score status remained an independent predictor of the composite endpoint, since it was associated with 77% higher chances of HF rehospitalization and all-cause mortality, even after adjusting for established outcome predictors, including the MAGGIC risk score, pharmacotherapy, and standard biochemical and echocardiographic parameters. This supports its utility for risk stratification and for identifying a high-risk subgroup that may need closer follow-up. Whether acting on the VExUS trajectory to guide decongestion or discharge timing improves outcomes was not tested in this observational study and requires further intervention evaluation.

Acute heart failure is a heterogeneous syndrome and the mechanisms of congestion may differ across etiologies. In our study, ischemic, dilated and hypertensive phenotypes were distributed comparably across VExUS trajectory groups, whereas valvular heart disease predominated among non-improved patients; this is consistent with the identification of severe tricuspid regurgitation as the principal determinant of non-improvement. Dedicated etiology-stratified outcome analyses were precluded by subgroup size and represent a direction for future multicenter studies.

### 4.1. Prognosis in Acute HF After Discharge: Beyond Conventional Markers

Prediction of clinical outcomes following discharge for acute HF hospitalization is of utmost clinical interest, as prompt identification of high-risk individuals is necessary for further interventions that extend event-free survival [14]. Traditionally, clinicians have relied on clinical parameters to guide management and decide the appropriate timing for discharge. Markers such as peripheral edema, jugular venous pressure, and the presence of a third heart sound have long been utilized as indicators of volume status. However, physical examination has been consistently shown to lack sufficient sensitivity to detect subclinical congestion [15] and suffers from limited reproducibility, owing to the subjective nature of clinical sign interpretation [16]. The MAGGIC risk score streamlines the process of applying clinical variables to gauge prognosis of HF, incorporating 13 clinical variables to provide a comprehensive risk profile [17]. However, while it offers valuable insight into long-term survival in the ambulatory setting, it is considered less sensitive to the vulnerable period immediately following discharge, where rehospitalization is primarily driven by residual congestion [18].

Biochemical markers, with NT-proBNP at the forefront, are also integral to risk assessment. Elevated levels of natriuretic peptides have been shown to have a robust correlation to both short- and long-term outcomes in HF patients, reflecting sustained myocardial wall stress [19,20,21]. However, NT-proBNP levels may be inflated by a number of confounders, including age, obesity, sex, and renal function [22], therefore limiting the marker’s reliability for volume status approximation. Conventional echocardiography provides structural and functional context to volume assessment. Right chamber parameters, such as RV end-diastolic area, RV free-wall longitudinal strain, and right atrial volume among others, demonstrate a strong correlation with clinical deterioration and cardiovascular events [23,24,25]. Integration of VExUS evolution from first contact to discharge assesses congestion in a dynamic and holistic manner, providing incremental value beyond the aforementioned modalities. The primary finding of the current study is the incremental value that VExUS non-improvement provides over these established models. While Model 2, which included the MAGGIC score, NT-proBNP, pharmacotherapy and comprehensive echocardiographic variables, showed significant prognostic power, the addition of the VExUS trajectory resulted in a further significant increase in model fit (Δχ^2^ = 9.22, *p* = 0.007). This indicates that persistent venous congestion at discharge, as captured by the flow patterns in the hepatic, portal, and renal veins, provides independent information that clinical risk scores and standard imaging cannot fully encompass.

### 4.2. VExUS Score: Utility in Clinical Applications

The VExUS protocol was originally validated as a tool to predict acute kidney injury in the intensive care setting [9]. Since its introduction, its application has shifted toward acute HF management, with most studies either capturing a single measurement during admission or discharge [26,27,28], or identifying early score reductions within the first 72 h of admission [29,30]. These findings confirmed that scores of 2–3 and a lack of early improvement have a strong correlation with in-hospital mortality and rehospitalization within up to 3 months of discharge [26,27,28,29]. This study adds an important dimension to the existing body of work by emphasizing VExUS score trajectory throughout the entire hospitalization, which identifies a substantial subgroup of patients with refractory subclinical congestion. At the time of discharge, these patients lack overt signs of volume excess, yet this modality is able to detect them, highlighting the need for improved decongestive strategies tailored to this subgroup. While the VExUS score has been shown to detect intravascular volume changes, surpassing lung ultrasound and Doppler E/e’ ratio [29], the present analysis clarifies the pathophysiological drivers of persistent congestion. By identifying severe tricuspid regurgitation as the sole independent predictor of non-improvement, it is suggested that persistent congestion is often due to mechanical causes needing extra specialized treatment, rather than a simple failure of diuretic titration. This “non-improved VExUS score” phenotype represents a state of hemodynamic resistance that remains invisible to traditional markers but provides significant incremental prognostic value beyond other, well-established prognostic variables.

## 5. Limitations

The present analysis is based on data from a single center which may limit the generalizability of the findings. Second, VExUS assessment relies on pulsed-wave Doppler interrogation of extracardiac veins and therefore requires specific operator expertise. Although all examinations in this study were performed by trained cardiologists with dedicated experience in venous Doppler assessment, only two operators from the echocardiography laboratory (representing 25% of staff) performed VExUS evaluations, which may constrain broader applicability. Other technical limitations of VExUS feasibility arose from factors such as elevated body mass index, limited patient cooperation, or suboptimal acoustic windows. Finally, this was an observational study, and causal inference is therefore limited. Despite multivariable adjustment, residual and unmeasured confounding cannot be excluded. Our findings establish the prognostic value of VExUS trajectory but do not demonstrate that VExUS-guided treatment or discharge decisions improve clinical outcomes, which would require a dedicated interventional trial.

## 6. Conclusions

Persistent systemic venous congestion, as quantified by serial VExUS score assessments, identifies a high-risk population with advanced hemodynamic compromise and adverse clinical profiles. Failure of VExUS to improve during hospitalization reflects not only unresolved congestion but also underlying structural and functional right heart abnormalities, with severe tricuspid regurgitation emerging as a key marker. VExUS score non-improvement was associated with worse event-free survival and provided incremental prognostic value beyond established clinical risk scores, laboratory biomarkers, and comprehensive echocardiographic assessment, underscoring its ability to refine risk stratification in acute HF. These findings highlight the potential utility of serial VExUS evaluation as a tool for detecting residual subclinical congestion and refining risk stratification in patients hospitalized for acute HF. Further studies are required to assess whether VExUS-guided decongestion or discharge decisions improve long-term outcomes.

## Figures and Tables

**Table 1:** Baseline clinical characteristics.

Variable	All (*n* = 418)	Non-Congested (*n* = 81)	Improved VExUS (*n* = 214)	Non-Improved VExUS (*n* = 123)	*p*-Value
**Clinical characteristics**					
Age, years	73.3 ± 11.8	72.5 ± 11.9	72.9 ± 12.2	74.7 ± 11.1	0.32
Male gender, *n* (%) Systolic blood pressure, mmHg Heart rate, bpm	246 (59.1%) 126.1 ± 19.1 83.0 ± 17.4	47 (58.0%) 126.3 ± 19.6 82.8 ± 16.5	132 (61.7%) 126.7 ± 19.8 84.8 ± 18.5	68 (55.3%) 125.0 ± 17.5 80.2 ± 15.9	0.50 0.75 0.11
NYHA class	2.2 ± 0.8	1.9 ± 0.8 ^^#^	2.12 ± 0.8 ^*	2.6 ± 0.8 ^#^*	<0.001
Body mass index, kg/m^2^	28.2 ± 5.5	28.0 ± 4.8	28.6 ± 5.9	27.6 ± 5.1	0.46
MAGGIC Risk Score	23.3 ± 7.8	21.0 ± 7.3 ^#^	22.7 ± 7.8 *	25.8 ± 7.7 ^#^*	<0.001
**Medical History**					
Heart failure diagnosis ≥ 18 months	219 (52.4%)	29 (35.8%) ^#^	108 (50.5%) *	82 (66.7%) ^#^*	<0.001
Smoking	82 (19.6%)	26 (32.1%) ^#^	45 (21.0%) *	11 (8.9%) ^#^*	<0.001
Ischemic heart disease, *n* (%)	152 (36.5%)	37 (45.7%)	76 (35.7%)	39 (31.7%)	0.12
Dilated cardiomyopathy, *n* (%)	49 (11.7%)	6 (7.4%)	28 (13.1%)	15 (12.2%)	0.393
Hypertension, *n* (%)	203 (28.8%)	44 (54.3%)	106 (50.0%)	53 (43.1%)	0.257
Type 2 diabetes mellitus, *n* (%)	159 (38.0%)	33 (40.7%)	79 (36.9%)	47 (38.2%)	0.83
COPD, *n* (%)	59 (14.1%)	11 (13.6%)	30 (14.0%)	18 (14.6%)	0.98
**Medication**					
ACEi/ARB	200 (47.8%)	49 (60.5%) ^#^	114 (53.3%) *	37 (30.1%) ^#^*	<0.001
Beta-blocker	350 (83.7%)	71 (87.7%)	179 (83.6%)	100 (81.3%)	0.48
Inotropes iv, *n* (%)	47 (11.2%)	4 (4.9%)	30 (14.0%)	13 (10.6%)	0.09
Furosemide iv dose, mg/day	154.22 ± 113.94	110.76 ± 87.62 ^^#^	157.53 ± 107.44 ^	178.60 ± 132.09 ^#^	<0.001
**Laboratory**					
NT-proBNP, pg/mL(range)	4376 (1881–9965)	2678 (1338–7401) ^^#^	5215 (2216–11,961) ^	4424 (2089–9883) ^#^	0.002
Creatitine, mg/dL	1.3 ± 0.5	1.2 ± 0.3 ^#^	1.3 ± 0.5 *	1.4 ± 0.6 *^#^	0.005

^ demonstrates statistically significant difference between non-congested and improved VExUS. ^#^ demonstrates statistically significant difference between non-congested and non-improved VExUS. * demonstrates statistically significant difference between improved VExUS and non-improved VExUS. Abbreviations: ACEi, angiotensin-converting enzyme inhibitor; ARB, angiotensin receptor blocker; COPD, chronic obstructive pulmonary disease; NT-proBNP, N-terminal pro B-type natriuretic peptide; NYHA, New York Heart Association; VExUS, venous excess ultrasound score.

**Table 2:** Baseline echocardiographic characteristics.

Variable	All (*n* = 418)	Non-Congested (*n* = 81)	Improved VExUS (*n* = 214)	Non-Improved VExUS (*n* = 123)	*p*-Value
**Left Ventricle**					
LV end-diastolic diameter, mm	55.1 ± 10.1	54.4 ± 10.5	55.3 ± 9.9	55.1 ± 10.2	0.93
LV end-systolic diameter, mm	44.3 ± 11.5	44.5 ± 11.0	44.6 ± 11.2	43.6 ± 12.4	0.44
LV end-diastolic volume indexed, mL/m^2^	85.0 ± 35.6	91.1 ± 34.2	84.1 ± 35.1	82.4 ± 37.4	0.06
LV end-systolic volume indexed, mL/m^2^	54.5 ± 32.4	59.5 ± 30.6	54.1 ± 31.6	51.8 ± 34.6	0.053
LV ejection fraction, %	39.9 ± 14.2	38.0 ± 12.5	39.4 ± 14.2	41.9 ± 15.0	0.12
LV global longitudinal strain, %	10.4 ± 5.0	9.0 ± 3.3 ^#^	10.3 ± 4.8 *	11.6 ± 6.1 *^#^	0.007
E, cm/s	103.0 ± 27.0	92.2 ± 27.1 ^^#^	103.8 ± 26.7 ^	109.1 ± 25.6 ^#^	<0.001
A, cm/s	61.0 ± 27.8	68.2 ± 29.7 ^^#^	58.2 ± 26.7 ^	58.0 ± 26.9 ^#^	0.048
Average E/e ratio	17.94 ± 5.47	18.45 ± 5.64	18.08 ± 5.40	17.34 ± 5.48	0.393
**Right Ventricle**					
RV end-diastolic area, cm^2^	21.6 ± 6.7	18.0 ± 5.0 ^^#^	21.4 ± 6.4 ^*	24.4 ± 7.1 *^#^	<0.001
RV end-systolic area, cm^2^	14.4 ± 5.7	11.3 ± 4.5 ^^#^	14.3 ± 5.4 ^*	16.7 ± 5.9 *^#^	<0.001
Fractional area change, %	34.3 ± 9.6	38.5 ± 9.7 ^^#^	33.9 ± 9.3 ^	32.3 ± 9.3 ^#^	<0.001
S’TDI tricuspid, cm/s	9.6 ± 3.0	10.9 ± 3.1 ^^#^	9.6 ± 2.8 ^*	8.8 ± 2.8 *^#^	<0.001
Tricuspid annular plane systolic excursion, mm	16.4 ± 4.1	18.1 ± 4.1 ^^#^	16.3 ± 3.9 ^	15.5 ± 4.1 ^#^	<0.001
Pulmonary valve acceleration time, ms	83.0 ± 23.3	88.8 ± 20.1 ^^#^	81.7 ± 22.5 ^	81.4 ± 26.0 ^#^	0.006
RV free wall longitudinal strain, %	19.5 ± 6.7	21.6 ± 6.5 ^#^	19.6 ± 6.7	18.1 ± 6.3 ^#^	0.001
**Others**					
Left atrial volume indexed, mL/m^2^	59.8 ± 29.1	49.4 ± 15.7 ^^#^	61.2 ± 34.1 ^	64.0 ± 24.7 ^#^	<0.001
Left atrial reservoir strain, %	8.8 ± 4.7	10.9 ± 4.7 ^^#^	8.4 ± 4.6 ^	8.2 ± 4.5 ^#^	<0.001
Right atrial volume, mL	83.6 ± 46.7	50.2 ± 22.7 ^^#^	83.0 ± 41.4 ^*	106.7 ± 53.2 *^#^	<0.001
Right atrial reservoir strain, %	14.4 ± 10.7	22.8 ± 10.9 ^^#^	13.7 ± 9.8 ^*	10.2 ± 9.0 *^#^	<0.001
Severe left-sided valvular heart disease, %	84 (20.1%)	11 (13.6%) ^#^	38 (17.8%)	35 (28.5%) ^#^	0.016
Severe tricuspid regurgitation, %	147 (35.2%)	5 (6.2%) ^^#^	65 (30.4%) ^*	77 (62.6%) *^#^	<0.001
RV systolic pressure, mmHg	48.8 ± 13.2	36.6 ± 8.9 ^^#^	50.4 ± 11.3 ^	54.1 ± 13.8 ^#^	<0.001
Inferior vena cava diameter, mm	22.8 ± 5.5	15.1 ± 3.1 ^^#^	24.0 ± 3.4 ^*	25.9 ± 5.2 *^#^	<0.001

^ demonstrates statistically significant difference between non-congested and improved VExUS. ^#^ demonstrates statistically significant difference between non-congested and non-improvedVExUS. * demonstrates statistically significant difference between improved VExUS and non-improvedVExUS. Abbreviations: LV, left ventricle; RV, right ventricle; TDI, tissue Doppler imaging; VExUS, venous excess ultrasound score.

**Table 3:** Predictors of non-improved VExUS score.

	Univariable			Multivariable		
Variable	OR	95% CI	*p*-Value	OR	95% CI	*p*-Value
MAGGIC Risk Score *	1.06	1.03–1.09	<0.001	1.03	0.99–1.07	0.12
NT-proBNP, pg/mL **	1.12	0.72–1.74	0.62			
LV end-diastolic volume indexed, mL/m^2^	0.99	0.99–1.00	0.35			
E, cm/s	1.01	1.00–1.02	0.005	1.01	0.99–1.02	0.27
Average E/e ratio	1.03	0.99–1.07	0.17			
RV end-diastolic area, cm^2^	1.09	1.06–1.13	<0.001	1.03	0.98–1.08	0.32
Tricuspid annular plane systolic excursion, mm ***	0.92	0.88–0.98	0.004			
Fractional area change, % ***	0.97	0.95–0.99	0.006			
S’TDI tricuspid, cm/s ***	0.86	0.80–0.93	<0.001			
RV free-wall longitudinal strain, % ***	0.95	0.92–0.99	0.004	0.98	0.93–1.03	0.44
Left atrial volume indexed, mL/m^2^	1.01	0.99–1.01	0.07			
Left atrial reservoir strain, %	0.96	0.91–1.01	0.08			
Right atrial volume, mL	1.02	1.01–1.02	<0.001	1.01	0.99–1.02	0.08
Right atrial reservoir strain, %	1.02	1.01–1.02	<0.001	1.00	0.97–1.04	0.84
Severe left-sided valvular heart disease	2.00	1.21–3.28	0.006	1.17	0.60–2.24	0.64
Severe tricuspid regurgitation	5.38	3.42–8.47	<0.001	2.86	1.51–5.45	0.001
Inotropes iv	0.91	0.46–1.79	0.78			
Furosemide iv dose, mg/day	1.00	1.00–1.00	0.007	1.00	0.98–1.00	0.73

* MAGGIC Risk Score incorporates the following variables: Age, LV Ejection Fraction, Systolic blood pressure, Body mass index, Creatinine, NYHA Class, Gender, Smoking, Diabetes, Chronic obstructive pulmonary disease, Heart failure first diagnosed ≥ 18 months, Beta-blocker, ACEi/ARB. ** The logarithmic transform of NT-proBNP was introduced in this regression analysis. *** RV free wall longitudinal strain was selected among the RV function indices to be included in the multivariable analysis as a novel, robust metric of RV function, to avoid collinearity. Abbreviations: ACEi, angiotensin-converting enzyme inhibitor; ARB, angiotensin receptor blocker; LV, left ventricle; NT-proBNP, N-terminal pro B-type natriuretic peptide; NYHA, New York Heart Association; RV, right ventricle; TDI, tissue Doppler imaging; VExUS, venous excess ultrasound score.

**Table 4:** Univariable and multivariable Cox regression analysis for composite primary endpoint.

	Univariable			Multivariable		
Variable	HR	95% CI	*p*-Value	HR	95% CI	*p*-Value
MAGGIC Risk Score *	1.07	1.05–1.09	<0.001	1.01	0.98–1.04	0.39
NT-proBNP, pg/mL **	2.07	1.49–2.90	<0.001	1.40	0.88–2.22	0.15
LV end-diastolic volume indexed, mL/m^2^	1.00	0.99–1.00	0.29			
LV end-systolic volume indexed, mL/m^2^	1.00	0.99–1.00	0.49			
LV global longitudinal strain, %	1.00	0.97–1.03	0.87			
E, cm/s	1.01	1.00–1.01	0.06			
Average E/e ratio	1.03	0.99–1.06	0.11			
RV end-diastolic area, cm^2^	1.03	1.01–1.05	0.006	0.98	0.94–1.01	0.20
Tricuspid annular plane systolic excursion, mm ***	0.91	0.87–0.95	<0.001			
Fractional area change, % ***	0.98	0.97–1.00	0.025			
S’TDI tricuspid, cm/s ***	0.87	0.92–0.93	<0.001			
RV free-wall longitudinal strain, % ***	0.97	0.95–0.99	0.012	1.01	0.98–1.06	0.48
Left atrial volume indexed, mL/m^2^	1.00	1.00–1.01	0.65			
Left atrial reservoir strain, %	0.96	0.93–1.00	0.050			
Right atrial volume, mL	1.01	1.00–1.01	<0.001	1.00	0.99–1.01	0.89
Right atrial reservoir strain, %	0.95	0.93–0.97	<0.001	0.98	0.95–1.01	0.10
RV systolic pressure, mmHg	1.02	1.01–1.03	<0.001	1.01	0.99–1.02	0.55
Severe left-sided valvular heart disease	1.59	1.10–2.28	0.013	1.15	0.73–1.81	0.54
Severe tricuspid regurgitation	3.00	2.18–4.13	<0.001	1.88	1.16–3.02	0.010
Inotropes intravenous	2.51	1.67–3.79	<0.001	1.13	0.65–1.96	0.68
Furosemide intravenous dose, mg/day	1.00	1.00–1.01	<0.001	1.00	1.00–1.01	0.011
Non-improved VExUS	2.43	1.76–3.34	<0.001	1.77	1.17–2.67	0.007

* MAGGIC Risk Score incorporates the following variables: Age, LV Ejection Fraction, Systolic blood pressure, Body mass index, Creatinine, NYHA Class, Gender, Smoking, Diabetes, Chronic obstructive pulmonary disease, Heart failure first diagnosed ≥ 18 months, Beta-blocker, ACEi/ARB. ** The logarithmic transform of NT-proBNP was introduced in this regression analysis. *** RV free wall longitudinal strain was selected among the RV function indices to be included in the multivariable analysis as a novel, robust metric of RV function, to avoid collinearity. Abbreviations: ACEi, angiotensin-converting enzyme inhibitor; ARB, angiotensin receptor blocker; LV, left ventricle; NT-proBNP, N-terminal pro B-type natriuretic peptide; NYHA, New York Heart Association; RV, right ventricle; TDI, tissue Doppler imaging; VExUS, venous excess ultrasound score.

## Data Availability

The raw data supporting the conclusions of this article will be made available by the authors upon request.

## Supplementary Materials

The following supporting information can be downloaded at https://www.mdpi.com/article/10.3390/diagnostics16203374/s1. Echocardiographic protocol. References [31,32,33,34,35] are cited in the Supplementary Materials.

## Abbreviations

IVC	Inferior Vena Cava
MAGGIC	Meta-Analysis Global Group in Chronic Heart Failure
NT-proBNP	N-terminal pro B-type natriuretic peptide
NYHA	New York Heart Association
VExUS	Venous Excess Ultrasound
HF	Heart Failure
RV	Right Ventricle
RA	Right atrium
E	early diastolic transmitral flow velocity
A	late (atrial) diastolic transmitral flow velocity
ACEi	Angiotensin-Converting Enzyme Inhibitor
ARB	Angiotensin Receptor Blocker

## References

[1] Rubio-Gracia J., Demissei B.G., ter Maaten J.M., Cleland J.G., O’Connor C.M., Metra M., Ponikowski P., Teerlink J.R., Cotter G., Davison B.A. (2018). Prevalence, predictors and clinical outcome of residual congestion in acute decompensated heart failure. Int. J. Cardiol..

[2] Magnino C., Omedè P., Avenatti E., Presutti D., Iannaccone A., Chiarlo M., Moretti C., Gaita F., Veglio F., Milan A. (2017). Inaccuracy of Right Atrial Pressure Estimates Through Inferior Vena Cava Indices. Am. J. Cardiol..

[3] Longino A., Martin K., Leyba K., Siegel G., Thai T.N., Riscinti M., Douglas I.S., Gill E., Burke J. (2024). Prospective Evaluation of Venous Excess Ultrasound for Estimation of Venous Congestion. Chest.

[4] Longino A., Martin K., Leyba K., Siegel G., Gill E., Douglas I.S., Burke J. (2024). Correlation between the VExUS score and right atrial pressure: A pilot prospective observational study. Crit. Care.

[5] Anastasiou V., Stavropoulou E., Peteinidou E., Nikolaidou A., Fardoulis E., Giannakoulas G., Karamitsos T., Delgado V., Ziakas A., Kamperidis V. (2026). Long-Term Prognostic Implications of Venous Excess Ultrasound Score in Acute Heart Failure. J. Am. Soc. Echocardiogr..

[6] Sierra A., Berrio-Becerra C.A., Santillan Herrera S., Gopar-Nieto R., Araiza-Garaygordobil D. (2026). Ultrasound-guided decongestion using the VExUS protocol enhances NT-proBNP reduction in acute decompensated heart failure. Eur. Heart J. Acute Cardiovasc. Care.

[7] Anastasiou V., Daios S., Moysidis D.V., Bazmpani M.A., Zegkos T., Karamitsos T., Makedou K., Savopoulos C., Efthimiadis G., Ziakas A. (2023). Clinical Value of Novel Echocardiographic Biomarkers Assessing Myocardial Work in Acute Heart Failure—Rationale and Design of the “Beyond Myo-HF Study”. Diagnostics.

[8] Huette P., Guinot P.G., Haye G., Moussa M.D., Beyls C., Guilbart M., Martineau L., Dupont H., Mahjoub Y., Abou-Arab O. (2021). Portal Vein Pulsatility as a Dynamic Marker of Venous Congestion Following Cardiac Surgery: An Interventional Study Using Positive End-Expiratory Pressure. J. Clin. Med..

[9] Beaubien-Souligny W., Rola P., Haycock K., Bouchard J., Lamarche Y., Spiegel R., Denault A.Y. (2020). Quantifying systemic congestion with Point-Of-Care ultrasound: Development of the venous excess ultrasound grading system. Ultrasound J..

[10] Rudski L.G., Lai W.W., Afilalo J., Hua L., Handschumacher M.D., Chandrasekaran K., Solomon S.D., Louie E.K., Schiller N.B. (2010). Guidelines for the Echocardiographic Assessment of the Right Heart in Adults: A Report from the American Society of Echocardiography: Endorsed by the European Association of Echocardiography, a registered branch of the European Society of Cardiology, and the Canadian Society of Echocardiography. J. Am. Soc. Echocardiogr..

[11] Koratala A., Reisinger N. (2022). Venous Excess Doppler Ultrasound for the Nephrologist: Pearls and Pitfalls. Kidney Med..

[12] Iida N., Seo Y., Sai S., Machino-Ohtsuka T., Yamamoto M., Ishizu T., Kawakami Y., Aonuma K. (2016). Clinical Implications of Intrarenal Hemodynamic Evaluation by Doppler Ultrasonography in Heart Failure. JACC Heart Fail..

[13] Rich J.D., Burns J., Freed B.H., Maurer M.S., Burkhoff D., Shah S.J. (2018). Meta-Analysis Global Group in Chronic (MAGGIC) Heart Failure Risk Score: Validation of a Simple Tool for the Prediction of Morbidity and Mortality in Heart Failure with Preserved Ejection Fraction. J. Am. Heart Assoc..

[14] Bozkurt B. (2023). Successful Decongestion as a Clinical Target, Performance Indicator, and as a Study Endpoint in Hospitalized Heart Failure Patients. JACC Heart Fail..

[15] Stevenson L.W., Perloff J.K. (1989). The Limited Reliability of Physical Signs for Estimating Hemodynamics in Chronic Heart Failure. JAMA.

[16] Stein J.H., Neumann A., Marcus R.H. (1997). Comparison of Estimates of Right Atrial Pressure by Physical Examination and Echocardiography in Patients with Congestive Heart Failure and Reasons for Discrepancies. Am. J. Cardiol..

[17] Pocock S.J., Ariti C.A., McMurray J.J.V., Maggioni A., Køber L., Squire I.B., Swedberg K., Dobson J., Poppe K.K., Whalley G.A. (2013). Predicting survival in heart failure: A risk score based on 39 372 patients from 30 studies. Eur. Heart J..

[18] Michaels A., Aurora L., Peterson E., Liu B., Pinto Y.M., Sabbah H.N., Williams K., Lanfear D.E. (2020). Risk Prediction in Transition: MAGGIC Score Performance at Discharge and Incremental Utility of Natriuretic Peptides. J. Card. Fail..

[19] Molvin J., Jujic A., Bachus E., Gallo W., Tasevska-Dinevska G., Holm H., Melander O., Fedorowski A., Magnusson M. (2019). Cardiovascular Biomarkers Predict Post-Discharge Re-Hospitalization Risk and Mortality Among Swedish Heart Failure Patients. ESC Heart Fail..

[20] Buchan T.A., Ching C., Foroutan F., Malik A., Daza J.F., Hing N.N.F., Siemieniuk R., Evaniew N., Orchanian-Cheff A., Ross H.J. (2022). Prognostic value of natriuretic peptides in heart failure: Systematic review and meta-analysis. Heart Fail. Rev..

[21] Noveanu M., Breidthardt T., Potocki M., Reichlin T., Twerenbold R., Uthoff H., Socrates T., Arenja N., Reiter M., Meissner J. (2011). Direct comparison of serial B-type natriuretic peptide and NT-proBNP levels for prediction of short- and long-term outcome in acute decompensated heart failure. Crit. Care.

[22] Basha M., Stavropoulou E., Nikolaidou A., Dividis G., Peteinidou E., Tsioufis P., Kamperidis N., Dimitriadis K., Karamitsos T., Giannakoulas G. (2025). Diagnosing Heart Failure with Preserved Ejection Fraction in Obese Patients. J. Clin. Med..

[23] Palazzuoli A., Ruocco G., Evangelista I., De Vivo O., Nuti R., Ghio S. (2020). Prognostic Significance of an Early Echocardiographic Evaluation of Right Ventricular Dimension and Function in Acute Heart Failure. J. Card. Fail..

[24] Hamada-Harimura Y., Seo Y., Ishizu T., Nishi I., Machino-Ohtsuka T., Yamamoto M., Sugano A., Sato K., Sai S., Obara K. (2018). Incremental Prognostic Value of Right Ventricular Strain in Patients with Acute Decompensated Heart Failure. Circ. Cardiovasc. Imaging.

[25] Gentile F., Chianca M., Bazan L., Sciarrone P., Chubuchny V., Taddei C., Poggianti E., Passino C., Emdin M., Giannoni A. (2025). Incremental Prognostic Value of Echocardiography Measures of Right Ventricular Systolic Function in Patients with Chronic Heart Failure. J. Am. Heart Assoc..

[26] Chaves V.B., Barbosa L.M., Oliveira V.M.R., de Almeida V.P., Silva R.P., Maciel L.G., Gomes M.F.C.F., Zawam Y., Nunes M.D.C.P. (2026). VEXUS protocol as a prognostic tool in acute heart failure: A systematic review and Bayesian meta-analysis. Curr. Probl. Cardiol..

[27] Rinaldi P.M., Rihl M.F., Boniatti M.M. (2024). VExUS Score at Discharge as a Predictor of Readmission in Patients with Acute Decompensated Heart Failure: A Cohort Study. Arq. Bras. Cardiol..

[28] Senay F.B., Kaya M., Atlanoglu S., Ulu M. (2025). Effectiveness of VExUS scores for predicting mortality among patients followed up in the emergency department due to congestive heart failure: A prospective cross-sectional study. BMC Emerg. Med..

[29] Saadi M.P., Silvano G.P., Machado G.P., Almeida R.F., Scolari F.L., Biolo A., Aboumarie H.S., Telo G.H., da Silveira A.D. (2025). Modified Venous Excess Ultrasound: A Dynamic Tool to Predict Mortality in Acute Decompensated Heart Failure. J. Am. Soc. Echocardiogr..

[30] Trigkidis K.K., Siempos I.I., Kotanidou A., Zakynthinos S., Routsi C., Kokkoris S. (2024). Early trajectory of venous excess ultrasound score is associated with clinical outcomes of general ICU patients. Shock.

[31] Lang R.M., Badano L.P., Mor-Avi V., Afilalo J., Armstrong A., Ernande L., Flachskampf F.A., Foster E., Goldstein S.A., Kuznetsova T. (2015). Recommendations for Cardiac Chamber Quantification by Echocardiography in Adults: An Update from the American Society of Echocardiography and the European Association of Cardiovascular Imaging. Eur. Heart J.-Cardiovasc. Imaging.

[32] Zoghbi W.A., Adams D., Bonow R.O., Enriquez-Sarano M., Foster E., Grayburn P.A., Hahn R.T., Han Y., Hung J., Lang R.M. (2017). Recommendations for Noninvasive Evaluation of Native Valvular Regurgitation. J. Am. Soc. Echocardiogr..

[33] Lancellotti P., Tribouilloy C., Hagendorff A., Popescu B.A., Edvardsen T., Pierard L.A., Badano L., Zamorano J.L. (2013). Recommendations for the echocardiographic assessment of native valvular regurgitation: An executive summary from the European Association of Cardiovascular Imaging. Eur. Heart J.-Cardiovasc. Imaging.

[34] Voigt J.-U., Pedrizzetti G., Lysyansky P., Marwick T.H., Houle H., Baumann R., Pedri S., Ito Y., Abe Y., Metz S. (2015). Definitions for a common standard for 2D speckle tracking echocardiography: Consensus document of the EACVI/ASE/Industry Task Force to standardize deformation imaging. Eur. Heart J.-Cardiovasc. Imaging.

[35] Badano L.P., Kolias T.J., Muraru D., Abraham T.P., Aurigemma G., Edvardsen T., D’Hooge J., Donal E., Fraser A.G., Marwick T. (2018). Standardization of left atrial, right ventricular, and right atrial deformation imaging using two-dimensional speckle tracking echocardiography: A consensus document of the EACVI/ASE/Industry Task Force to standardize deformation imaging. Eur. Heart J.-Cardiovasc. Imaging.

