# Supplementary material for: Determinants and Prognostic Value of Persistent Congestion Assessed by Venous Excess Ultrasound Score in Acute Heart Failure
Source: Diagnostics (Basel). 2026 Oct 16;16(20):3374. doi: 10.3390/diagnostics16203374 (PMC13703738; doi:10.3390/diagnostics16203374)
Supplement: Supplementary file 1 [file diagnostics-16-03374-s001.zip › diagnostics-4550555-supplementary.pdf]

SUPPLEMENTARY MATERIAL

Echocardiographic protocol

Left-sided assessment

Image acquisition followed the contemporary guideline recommendations and included M-mode, two-dimensional, colour, continuous, and Pulsed-Wave Doppler data were acquired, according to most recent recommendations [31–33]. For patients in atrial fibrillation, three consecutive beats were acquired and averaged for each measurement. Left ventricular (LV) ejection fraction was calculated via Simpson's biplane method. Left atrial (LA) maximum volume was obtained at end-systole from on the apical four- and two-chamber view using the biplane method and indexed to body surface area [31]. Evaluation of LV diastolic function comprised mitral inflow together with annular velocities from which the mean of the septal and lateral early diastolic annular velocities were used to derive the E/e' ratio. Left-sided valvular disease was interrogated via a multiparametric approach and severe valvular disease was classified in accordance with the corresponding guidelines [33].

Right-sided assessment

The right ventricle (RV) was assessed from a dedicated RV-focused apical view RV systolic function was characterized based on the guideline-recommended parameters including fractional area change (FAC), tricuspid annular plane systolic excursion (TAPSE) and the peak systolic velocity lateral tricuspid annulus (S') [10]. RV FAC was obtained by planimetry of the RV end-diastolic and end-systolic areas, TAPSE from M-mode assessment of the lateral annulus and S' from Pulse-Wave Tissue Doppler Imaging at the same location [10]. RV dimensions were recorded on an end-diastolic frame at the tricuspid annulus, at the RV base, at the mid-cavity and along the basal-to-apical long axis oriented perpendicular to the aforementioned measurements

[10]. The RV inflow E wave was measured by sampling the Pulse-Wave Doppler at the the tips of the tricuspid annulus and pulmonary valve acceleration time with Pulse-Wave Doppler at the tips of the pulmonary valve leaflets from a pulmonary artery-focused short-axis view [10]. Maximal right atrial (RA) volume was traced at end-systole on the same RV-focused view [10]. Tricuspid regurgitation was quantified using an integrative, multiparametric assessment and graded according to current guidelines[32,33]. Pulmonary artery systolic pressure was estimated from the peak tricuspid regurgitant velocity through the modified Bernoulli equation, with the addition of right atrial pressure inferred from the end-expiratory diameter and inspiratory collapse of the inferior vena cava [10].

Two-dimensional speckle-tracking echocardiographic analysis

All deformation indices are presented as absolute (positive) values. Speckle tracking analysis was carried out at frame rates of 50-80 Hz, averaging three cardiac cycles with the onset of the QRS complex taken as the reference point. LV global longitudinal strain (GLS) was calculated from three LV focused apical views, a four-chamber, a two-chanber, and a three-chamber view [34]. The region of interest was defined by the software and subsequently refined manually to cover the entire myocardium. Next, LV was divided into a 16-segment model and LV GLS was expressed as the mean of the peak values of all segments.

RV strain was derived from an apical RV-focused view based on a six-segment model that combined the basal, mid, and apical segments of the RV free wall with the basal, mid and apical septum [35]. RV free wall longitudinal strain was calculated as the mean of the RV basal, mid, and apical free-wall segments, with all septal segments

excluded [35]. Regions of interest were generated automatically and then manually adapted to the myocardial thickness of the RV.

For LA strain, the LA endocardial wall was traced manually in the apical four-chamber and two-chamber views and adjusted as needed to follow the atrial wall in detail. The software then divided the myocardium into six segments and strain was reported as the mean of the peak values of all segments. RA strain was assessed with the software dedicated to LA strain, applied to a manual trace that was obtained from the apical RV-focused view, following the same strategy. Reservoir strain was reported for both atria as it could be equally assessed for patients with atrial fibrillation.

1. Lang, R.M.; Badano, L.P.; Mor-Avi, V.; Afilalo, J.; Armstrong, A.; Ernande, L.; Flachskampf, F.A.; Foster, E.; Goldstein, S.A.; Kuznetsova, T.; et al. Recommendations for Cardiac Chamber Quantification by Echocardiography in Adults: An Update from the American Society of Echocardiography and the European Association of Cardiovascular Imaging. Eur. Heart J. Cardiovasc. Imaging 2015, 16, 233–271, doi:10.1093/ehjci/jev014.
2. Zoghbi, W.A.; Adams, D.; Bonow, R.O.; Enriquez-Sarano, M.; Foster, E.; Grayburn, P.A.; Hahn, R.T.; Han, Y.; Hung, J.; Lang, R.M.; et al. Recommendations for Noninvasive Evaluation of Native Valvular Regurgitation. J. Am. Soc. Echocardiogr. 2017, 30, 303–371, doi:10.1016/j.echo.2017.01.007.
3. Lancellotti, P.; Tribouilloy, C.; Hagendorff, A.; Popescu, B.A.; Edvardsen, T.; Pierard, L.A.; Badano, L.; Zamorano, J.L. Recommendations for the echocardiographic assessment of native valvular regurgitation: an executive summary from the European Association of Cardiovascular Imaging. Eur. Heart J. Cardiovasc. Imaging 2013, 14, 611–644, https://doi.org/10.1093/ehjci/jet105.
4. Voigt, J.-U.; Pedrizzetti, G.; Lysyansky, P.; Marwick, T.H.; Houle, H.; Baumann, R.; Pedri, S.; Ito, Y.; Abe, Y.; Metz, S.; et al. Definitions for a common standard for 2D speckle tracking echocardiography: consensus document of the EACVI/ASE/Industry Task Force to standardize deformation imaging. Eur. Hear. J. - Cardiovasc. Imaging 2015, 16, 1–11, https://doi.org/10.1093/ehjci/jeu184.
5. Badano, L.P.; Kolias, T.J.; Muraru, D.; Abraham, T.P.; Aurigemma, G.; Edvardsen, T.; D'Hooge, J.; Donal, E.; Fraser, A.G.; Marwick, T.; et al. Standardization of left atrial, right ventricular, and right atrial deformation imaging using two-dimensional speckle tracking echocardiography: a consensus document of the EACVI/ASE/Industry Task Force to standardize deformation imaging. Eur. Heart J. Cardiovasc. Imaging 2018, 19, 591–600, doi:10.1093/ehjci/jey042.
